# Lung adenocarcinoma with tumor resolution and dystrophic calcification after salvage surgery following immune checkpoint inhibitor therapy: A case report

**DOI:** 10.1111/1759-7714.13663

**Published:** 2020-09-15

**Authors:** Toshiyuki Sumi, Hirofumi Uehara, Toshiaki Masaoka, Makoto Tada, Yoshiko Keira, Koki Kamada, Naoki Shijubou, Yuichi Yamada, Hisashi Nakata, Yuji Mori, Hirofumi Chiba

**Affiliations:** ^1^ Department of Pulmonary Medicine Hakodate Goryoukaku Hospital Hakodate Japan; ^2^ Department of Respiratory Medicine and Allergology Sapporo Medical University School of Medicine Sapporo Japan; ^3^ Department of Thoracic Surgery Hakodate Goryoukaku Hospital Hakodate Japan; ^4^ Department of Surgical Pathology Hakodate Goryoukaku Hospital Hakodate Japan

**Keywords:** Adenocarcinoma, pembrolizumab, salvage therapy

## Abstract

A clinical trial of immune checkpoint inhibitors for advanced non‐small cell lung cancer reported an overall survival plateau with a long tail to the survival curve, suggesting that immune checkpoint inhibitors prolong survival. However, little evidence supports the efficacy of immune checkpoint inhibitors as neoadjuvant chemotherapy. We performed salvage surgery on a patient who was treated with an anti‐programmed cell death protein‐1 (PD‐1) antibody and whose tumor size had not changed over time. A 69‐year‐old Japanese female with advanced lung adenocarcinoma was initially administered pembrolizumab therapy; however, owing to the development of various immune‐related adverse events (irAEs), the patient was switched to chemotherapy following steroid therapy. The tumor continued to shrink and calcification within the tumor increased. We performed salvage surgery following which the tumor cells disappeared and necrosis and calcification were detected in the tumor. We concluded that if calcification develops within the tumor and tumor shrinkage is maintained after treatment with anti‐PD‐1 drugs, the calcification may be dystrophic owing to drug‐induced tumor necrosis, and salvage surgery might be beneficial in removing the tumor.

**Key points:**

**Significant findings of the study:**

- If calcification develops within the tumor and tumor shrinkage is maintained after treatment with anti‐PD‐1 drugs, the calcification may be dystrophic owing to tumor necrosis caused by drug effects, and salvage surgery might be beneficial in removing the tumor.

**What this study adds:**

- This study showed the efficacy of immune checkpoint inhibitors as neoadjuvant chemotherapy to be followed by salvage surgery for unresectable advanced lung adenocarcinoma.

## Graphical abstract

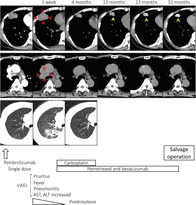

## Introduction

An anti‐programmed cell death protein‐1 (PD‐1) inhibitor, pembrolizumab, and pembrolizumab in combination with chemotherapy are frequently used as first‐line treatment in patients with non‐small cell lung cancer (NSCLC) because they exhibit longer progression‐free survival (PFS) and overall survival than chemotherapy alone.^1^, ^2^, ^3^, ^4^ A long‐term follow‐up clinical trial of a similar anti‐PD‐1 inhibitor, nivolumab, reported an overall survival plateau with a long tail to the survival curve,^5^ suggesting that immune checkpoint inhibitors prolong survival. However, determining whether a patient with advanced NSCLC is cured based on imaging alone is difficult. Few studies report the efficacy of immune checkpoint inhibitors as neoadjuvant chemotherapy.^6^, ^7^ We performed salvage surgery for unresectable advanced lung adenocarcinoma after initial chemotherapy with pembrolizumab because the tumor had shrunk and was responding to long‐term therapy.

## Case report

**Figure 1:**
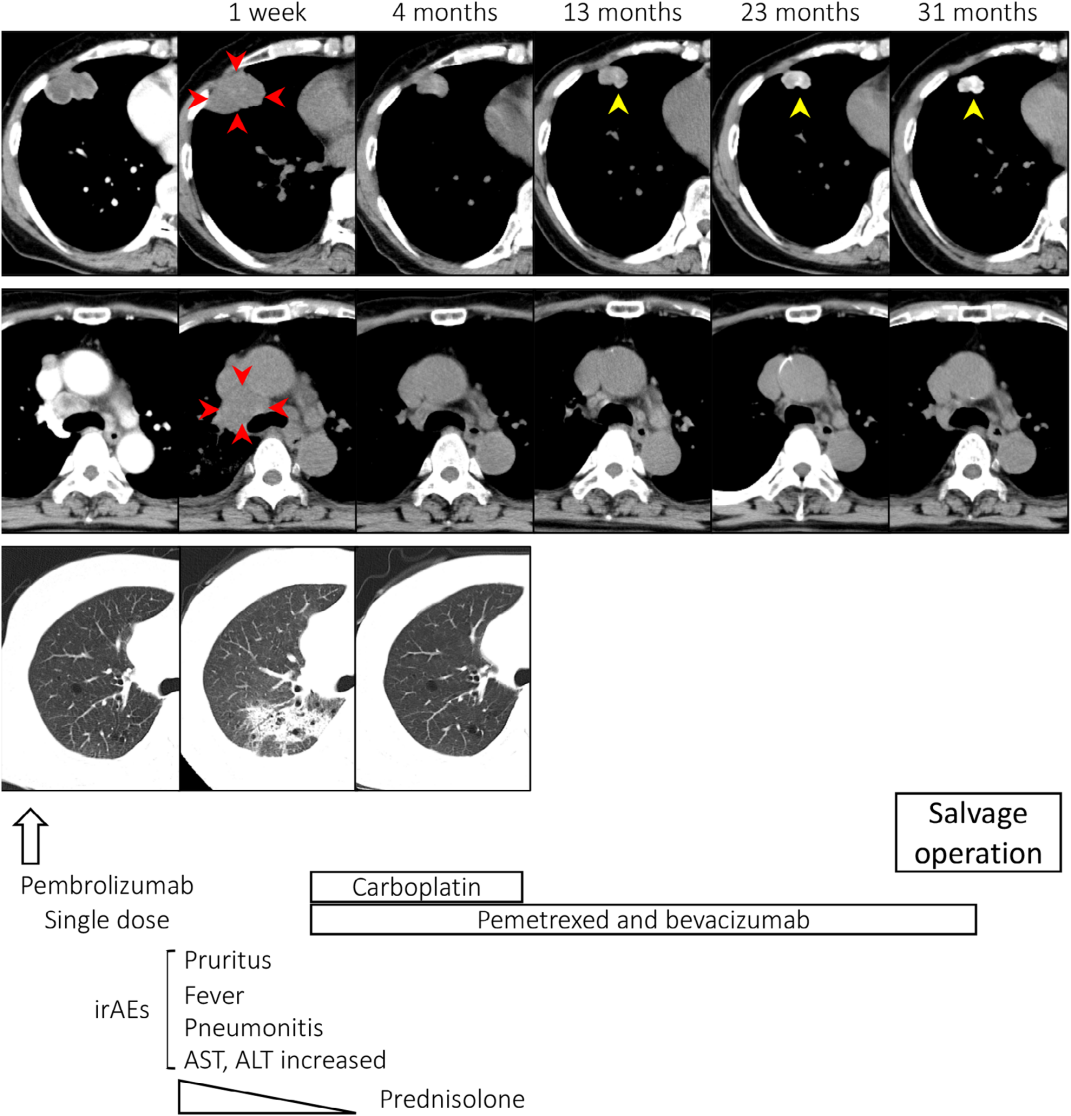
Clinical course. At one week, computed tomography (CT) showed enlarged primary tumor and increased mediastinal lymphadenopathy (red arrowhead), and interstitial lung disease (ILD) was apparent in the right lung upper field. Calcification within the primary tumor increased (yellow arrowhead).

**Figure 2:**
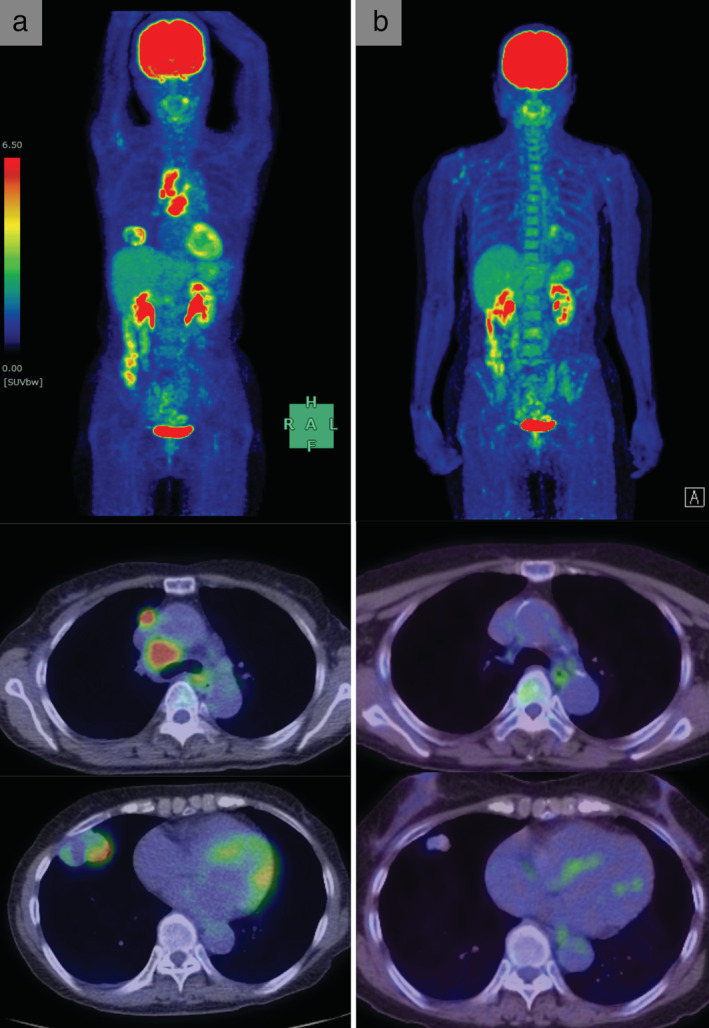
Fluorine‐18‐deoxyglucose (FDG)‐positron emission tomography imaging. (**a**) Before treatment. FDG accumulated in primary tumor and mediastinal lymph node metastasis. (**b**) After chemotherapy. FDG accumulation was only slightly observed at the primary tumor site.

**Figure 3:**
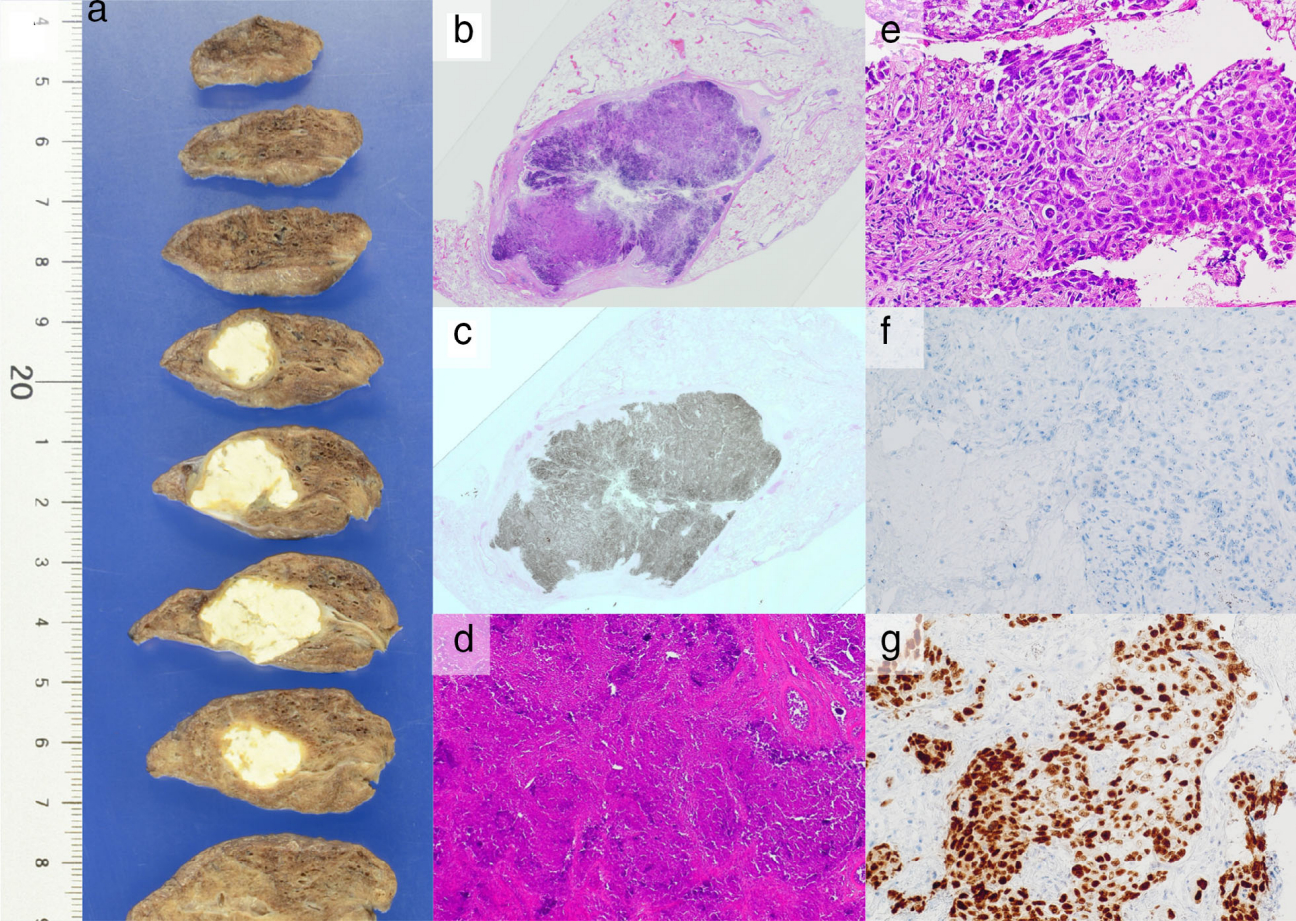
Pathological examinations. (**a**) Macroscopic findings. (**b**) Hematoxylin‐eosin stain: Loupe image. (**c**) Calcium deposits throughout the entire tumor. von Kossa stain reaction, Loupe image. (**d**) Coagulative necrosis tissue in the center of the tumor hematoxylin‐eosin stain × 10. (**e**) Transbronchial biopsy (TBB) specimen at diagnosis showed tumor cells hematoxylin‐eosin stain × 40. (**f**) Negative for p40 in TBB specimen. (**g**) Positive for thyroid transcription factor 1 in the TBB specimen.

A 69‐year‐old Japanese female, an ex‐smoker for 20 pack‐years with Eastern Cooperative Oncology Group performance status 0, was diagnosed with advanced lung adenocarcinoma at stage cT2a N2 M0. Driver mutations were negative, and the tumor proportion score was 95%. She was treated with pembrolizumab as first‐line chemotherapy, instead of chemoradiotherapy and surgery. The tumor was considered difficult to treat with radical radiation therapy as a large radiation field was required. Surgery was also ruled out owing to bulky N2 lymph node metastasis. Common Terminology Criteria for Adverse Events grade 2 pruritus was observed two days after administration, grade 2 fever was observed on day 3, and grade 3 liver dysfunction (increased aspartate transaminase and alanine transaminase) was observed on day 4. The grade 2 fever persisted and grade 3 pneumonitis (interstitial lung disease [ILD]) appeared on day 7. Computed tomography (CT) showed an enlarged primary tumor and enlarged mediastinal lymph nodes (Fig 1, red arrow). Prednisolone (1 mg/kg) was administered to treat ILD. As the fever and ILD disappeared, prednisolone was tapered off after two months. Continuation of pembrolizumab was determined to be a high risk for immune‐related adverse events (irAEs); therefore, carboplatin, pemetrexed, and bevacizumab therapy was initiated. At the end of four courses, the lymphadenopathy and the primary tumor had shrunk. Subsequently, maintenance therapy with pemetrexed and bevacizumab was continued. During maintenance therapy, calcification appeared inside the primary tumor on the CT scan and gradually increased (Fig 1, yellow arrow). The patient's calcium, phosphorus, parathyroid hormone, and vitamin D3 levels were in the normal range. Her sputum samples tested negative for mycobacterium, and interferon‐gamma release assays returned negative results; therefore, tuberculosis was ruled out. Magnetic resonance imaging showed no brain metastases, positron emission tomography/CT (PET/CT) revealed fluorine‐18‐deoxyglucose accumulation only in the primary site, and diseases other than the primary lesion were under control (Fig 2). Informed consent was obtained from the patient and salvage surgery was performed to resect the primary tumor. The removed tumor showed central coagulative necrosis and calcification without viable tumor cells (Fig 3). Chemotherapy was discontinued and the patient is now relapse‐free three years after receiving pembrolizumab.

## Discussion

We performed salvage surgery on a patient who was treated with a PD‐1 inhibitor and whose tumor size had not changed over time. After surgery, the primary tumor turned into nearly necrotic tissue, calcification was observed, and no residual tumor was confirmed.

The relationship between irAEs and therapeutic effect in anti‐PD‐1 inhibitor therapy may be associated with a good prognosis.^8^ Early irAEs are associated with a better outcome after treatment with immunotherapy.^9^ Moreover, patients showing pseudoprogression have significantly longer overall survival than patients showing typical progression.^10^ Although the patient was treated with pembrolizumab followed by chemotherapy, after the first dose of pembrolizumab, various irAEs developed early in the post‐treatment period and the tumor shrank after possible pseudoprogression. Therefore, just a single dose of pembrolizumab may have led to the acquisition of tumor immunity. The patient was treated with a single dose of pembrolizumab followed by chemotherapy with carboplatin, pemetrexed, and bevacizumab. In the AVAPERL study, the PFS of platinum, pemetrexed, and bevacizumab was 10.2 months and that over 18 months was less than 10%.^11^ Therefore, a long‐term response to single‐dose pembrolizumab was more likely than a sustained response to chemotherapy.

In this case, calcification increased in the primary tumor site over time. The patient did not have renal dysfunction or hypercalcemia associated with hyperparathyroidism. Additionally, only the central area of the tumor was necrotic, and calcification was consistent with the tumor site. The phenomenon of calcium deposition on degenerative necrotic tissue, even in the absence of hypercalcemia, is called dystrophic calcification.^12^, ^13^ Although the appearance of intratumor calcification during cetuximab plus chemotherapy in patients with metastatic colorectal cancer may be a positive prognostic factor,^14^ the relationship between tumor calcification and prognosis during chemotherapy in patients with lung cancer is still unclear. Pathological findings of this patient suggest that calcium is deposited on degenerated and necrotic tumor cells in response to immune checkpoint inhibitors and chemotherapy.

In conclusion, if calcification develops within the tumor and tumor shrinkage is maintained after treatment with anti‐PD‐1 drugs, the calcification may be dystrophic owing to tumor necrosis in response to the drug, and salvage surgery might be beneficial in removing the tumor.

## Disclosure

The authors declare that they have no competing interests. This research did not receive any specific grant from funding agencies in the public, commercial or not‐for‐profit sectors.
